# Long-Term Outcomes of Early Enzyme Replacement Therapy for Mucopolysaccharidosis IV: Clinical Case Studies of Two Siblings

**DOI:** 10.3390/diagnostics10020108

**Published:** 2020-02-17

**Authors:** Sharon Barak, Yair Anikster, Ifat Sarouk, Eve Stern, Etzyona Eisenstein, Tamar Yissar, Nir Sherr-Lurie, Annick Raas-Rothschild, Dafna Guttman

**Affiliations:** 1 Department of Pediatric Rehabilitation, Edmond and Lily Safra Children’s Hospital, Chaim Sheba Medical Center, Ramat-Gan 5265601, Israel; Etzyona.Eisenstein@sheba.health.gov.il (E.E.); Tamar.Yissar@sheba.health.gov (T.Y.); Dafna.Gutman@sheba.health.gov.il (D.G.); 2 Kaye Academic College of Education, M.Ed. programs, Beer-Sheva 8414201, Israel; 3 The Sackler School of Medicine, Tel Aviv University, Tel Aviv 6997801, Israel; Yair.Anikster@sheba.gov.il; 4 Edmond and Lily Safra Children’s Hospital, Chaim Sheba Medical Center, Ramat Gan 5265601, Israel; 5 Wohl Institute for Translational Medicine, Chaim Sheba Medical Center, Ramat Gan 5265601, Israel; 6 The National AT Center, Chaim Sheba Medical Center, Ramat Gan 5265601, Israel; Ifat.Sarouk@sheba.gov.il; 7 The Pediatric Pulmonology Unit, Chaim Sheba Medical Center, Edmond and Lilly Safra Children Hospital, Tel HaShomer, Ramat Gan 5265601, Israel; 8 Pediatric Endocrine and Diabetes Unit, Chaim Sheba Medical Center, Edmond and Lily Safra Children’s Hospital, Ramat-Gan 5265601, Israel; zipporaheve.stern@sheba.health.gov.il; 9 Pediatric Orthopedic Unit, Edmond and Lilly Safra Children Hospital, Chaim Sheba Medical Center, Ramat Gan 5265601, Israel; Nir.Sherr@sheba.health.gov.il; 10 Institute of Rare Diseases, Edmond and Lily Safra Children’s hospital, Chaim Sheba Medical Center, Ramat Gan 5265601, Israel; Annick.Rothschild@sheba.health.gov.il; 11 The Sackler Faculty of Medicine, Tel Aviv University, Tel Aviv 6997801, Israel

**Keywords:** case report, enzyme replacement therapy, glycosaminoglycan, deficient N-acetylgalactosamine 6-sulfatase, mucopolysaccharidosis, Morquio syndrome

## Abstract

Enzyme replacement therapy (ERT) is one of the available therapies for mucopolysaccharidosis (MPS). This study presents a follow-up of two siblings with MPS IVA (Morquio A disease) that received ERT. Both siblings received weekly intravenous infusions of elosulfase alfa for 4.5 years. One sibling (patient 1, P1; male) started therapy at 54 months of age, and the other sibling (patient 2, P2; female) started at 11 months of age. ERT was well-tolerated. In comparison to P1, P2’s growth curves deviated less from the norm. The orthopedic deformities of P1 were more severe than those of P2 and required several surgical corrections. P1’s sleep test at 48 months revealed obstructive sleep apnea, while by the age of 102 months, parameters were normal. P2 never had sleep apnea. Only P1 demonstrated ear, nose, and throat clinical illnesses. In comparison to P1, P2’s physical function was better maintained. In conclusion, ERT was safe in both patients during a 4.5-year follow-up. Although the typical characteristics of this disease were similar in both patients, P1 had a complex clinical course in comparison to P2, which influenced function and quality of life. Therefore, in order to make the most of ERT, it may be more beneficial when initiated at a relatively young age.

## 1. Introduction

Mucopolysaccharidosis (MPS) IVA (Morquio A disease) is one of a family of progressive genetic disorders that are caused by defects in glycosaminoglycan (GAG) catabolism, resulting in accumulation within lysosomes of partially degraded GAG [1]. The disease is caused by mutations in the gene encoding the lysosomal enzyme N-acetylgalactosamine 6-sulfatase (GALNS) [2,3,4]. The reduced GALNS activity causes an impairment in the catabolism of two GAGs, namely, keratan sulfate [5] and chondroitin-6-sulfate [6]. The distribution of GAGs in tissues is evident in the disease’s clinical presentation and mainly located in cartilaginous, bony, and muscular tissues [2,3,7].

Children with MPS IVA appear normal at birth [8] and by the end of the second year of life commonly present multiorgan disease involvement (e.g., skeletal and pulmonary), requiring complex multi-disciplinary medical attention [9,10]. The search for the optimal definitive treatment is still under investigation and currently consists of hematopoietic stem cell transplantation, gene therapy, and enzyme replacement therapy (ERT) [11].

Hematopoietic stem cell transplantation has proven to be a viable treatment option for patients with MPS types I, II, IVA, VI, and VII [12]. However, there is a need for more research to better understand this treatment in patients with MPS IVA [12]. Another treatment modality is gene therapy. The goal of gene therapy is to correct the genetic defect by direct insertion of normal deoxyribonucleic acid into the affected cells in order to institute endogenous production of the deficient enzyme by these cells. Findings on the effectiveness of gene therapy are encouraging, but additional work pertaining to immune reaction, choice of vector, and optimal route of administration is needed [13]. Finally, in 2014, ERT for MPS IVA using recombinant human GALNS, referred to as elosulfase alfa, was approved [14]. ERT is a lifelong appropriate therapy for some types of MPS with the purpose of reducing GAG accumulation [15,16,17,18,19]. Clinical trials show that ERT can improve quality of life and clinical and physical function, although eventually in the longterm, improvement reaches a plateau [20,21]. Overall, the current literature supports the safety and efficacy of ERT [22], especially when started at a young age [23,24,25,26].

There is a lack of long-term clinical studies that evaluate safety and efficacy of the use of ERT for MPS IVA. Moreover, there are studies showing that ERT for patients with MPS IVA might not always be useful. For example, according to Do Cao et al. [27] and Doherty et al. [28], early ERT did not improve skeletal outcomes in a patient with severe MPS IVA. Until now, there has been no proof that ERT has an impact on bone lesions. Still, according to Akyol et al. [29], the data are mainly limited to ERT among patients who initiated ERT relatively late into their disease progression. The limited data may cause reimbursement companies and physicians to not support ERT in relatively young children [30]. As the early initiation of ERT will likely have an effect on the course of the disease, there is a need for additional longitudinal observational studies of patients with this condition [11]. The purpose of this study is to present an approximately 4.5-year follow-up of two siblings with MPS IVA after initiation of ERT. One sibling (patient 1, P1; male) started therapy before the age of 5 years old, and the other sibling (patient 2, P2; female) started therapy at a younger age, before the age of 1 year old.

## 2. Materials and Methods

### 2.1. Selection of Subjects

Two siblings, a boy (P1) and a girl (P2) with MPS IVA that had started ERT at a young age, were identified from a Pediatric Rehabilitation Department. P1 was diagnosed when he was 49 months of age. P2, the younger sister of P1, was diagnosed after her brother at six months of age. Informed consent was obtained from the children’s parents. The study was approved (study code - 6625-19-SMC ; approval date – January 29, 2020) by the ethics committee of the Chaim Sheba Medical Center, Tel Hashomer, Israel.

### 2.2. Outcome Measures

Once diagnosed, the patients underwent regular assessments to evaluate the severity and progression of the disease. All data were retrospectively retrieved from the patients’ medical records and the treating physicians. The following is a description of the assessed outcome measures.

- Safety and compliance—safety evaluations included continuous monitoring of adverse events. Absences from treatment sessions were recorded.
- Hospitalization and surgeries history—data regarding the surgical and hospitalization (inpatient and outpatient) history of P1 and P2 were retrieved from medical records.
- Growth—height and weight were measured in routine visitations to the clinic.
- Orthopedic and radiographic assessments and procedures—orthopedic and radiographic assessments were routinely conducted in order to assess structural changes. As recommended, the radiographic assessments mainly focused on the lower extremities (e.g., presence of progressive hip dysplasia, genu valgus, and ankle valgus), upper extremities, cervical spine, and thoracolumbar spine [31]. All orthopedic procedures were documented.
- Respiratory function and sleep test—the respiratory function evaluation consisted of a pediatric pulmonologist physical examination, spirometry test (forced vital capacity, forced expiratory volume in one second), and oxygen saturation and overnight sleep study.
- Ear, nose, and throat (ENT) manifestations—patients underwent a routine evaluation by an otorhinolaryngologist.
- Physical function—evaluation of physical function was routinely conducted by physical and occupational therapists. The evaluation focused on the patient’s impairment levels (e.g., muscle strength, range of motion), mobility ability (e.g., walking and stair climbing ability), activities of daily living (e.g., offing and doffing), and equilibrium and protective reactions.

## 3. Results

P1 was diagnosed with MPS IVA at age 49 months and P2 at six months of age, after her brother’s diagnosis. Both were found to be compound heterozygote for G139S and R386C. Upon diagnosis, GALNS activity for both patients was 1.1 nmol/h/mg. Current GALNS activity is 0.1 micromol/L/h (cut-off value > 0.2). P1 and P2 started ERT with elosulfase alfa (VIMIZIM^®^ by BioMarin©) 5 months post-diagnosis at age 4.5 years and 11 months, respectively, with an intravenous dose of 1.0 mg/kg/week. The follow-up period of ERT is 4.5 years, whereas overall data include an additional 19 months prior to diagnosis for P1.

### 3.1. Safety and Compliance

Neither sibling has experienced any drug-related adverse events or infusion-associated reactions. Compliance with treatment was very good for both, with less than 10% of the programmed infusions missed per year.

### 3.2. Growth

**Figure 1:**
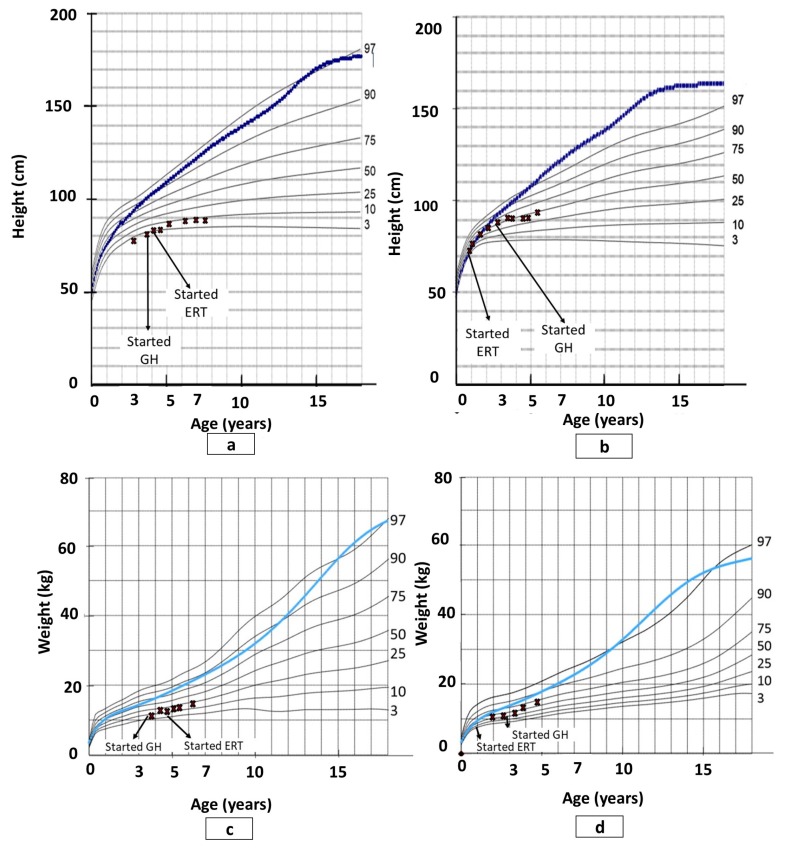
Growth charts of males and females with mucopolysaccharidosis IVA (MPS IVA) and study participants: (**a**) patient 1 height; (**b**) patient 2 height; (**c**) patient 1 weight; (**d**) patient 2 weight. Note: ERT, enzyme replacement therapy; GH, growth hormone; the dotted and blue lines show the 50th percentile values for normal males and females; the Xs show study participants; revised from Montaño et al. [32].

**Figure 2:**
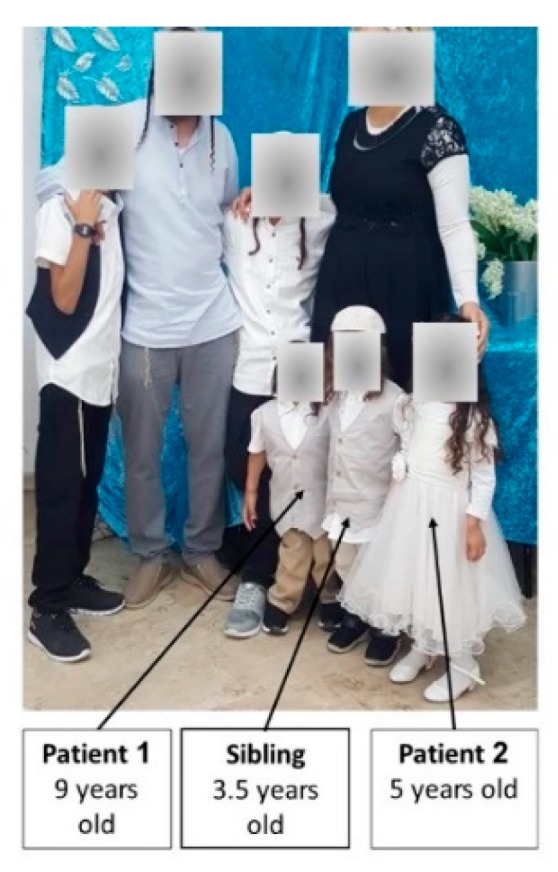
Height of patients 1 and 2, and their sibling.

P1 had been under pediatric endocrinology follow-up from age 18 months due to short stature and started growth hormone (GH) therapy at the age of 43 months for idiopathic short stature. Initially, he had a good response to the therapy with a height velocity of 4.2 cm in the first year following treatment initiation, compared with 0 cm in the prior year. GH therapy continued for 5 years and then discontinued because of growth failure and even height loss. P1 did grow somewhat once ERT was started; however, his height remained below the 10th percentile and his weight increased from the 10th to the 25th percentile of patients with MPS IVA. P2’s height and weight were at the 50th percentile of the normal population up to the age of 24 months, then significantly decreased, requiring the use of MPS IVA population curves [32]. On those curves, her height stabilized on the 50th percentile and weight on the 75th percentile until the end of the follow-up period (see Figure 1). Figure 2 shows the height differences between P1, P2, and their sibling.

### 3.3. Clinical Course

P1 was first admitted to the Pediatric Intensive Care Unit at the age of 30 months because of high cervical spine subluxation with cord contusion. Odontoid hypoplasia was noted on consecutive radiological studies. Treatment was conservative, using a neck and chest brace 24 h/d and an extended rehabilitation process. The brace was used for 4 years and then removed one year after he had C1-C2 posterior spinal fusion. By that time, he remained with mild left upper extremity monoparesis and overall muscular wasting and weakness. P1 underwent his second extended orthopedic surgery, which consisted of a shelf procedure for both hips, proximal femoral osteotomies, and insertion of eight plates for bilateral genu-valgus repair at 84 months of age, 30 months after ERT initiation. Subsequently, P1 received 6 months of daycare therapy in a rehabilitation center. Surgical procedures were conducted in a specialized center abroad. P1 received 18 months of ERT at the Pediatric Intensive Care Unit because of his fragile clinical status. Subsequent treatments were given at home. P1 has had regular weekly multiple therapies in a community setting since the age of 3 years (36 months) including physical therapy, occupational therapy, hydrotherapy, and psychological therapy.

In all follow-up years, P2 did not require surgeries and received ERT at the pediatric daycare department for 18 months. Following this, ERT was continued at home. P2 started community-based physical and occupational therapy sessions at the age of 4.5 years old.

### 3.4. Orthopedic and Radiographic Assessments and Procedures

**Figure 3:**
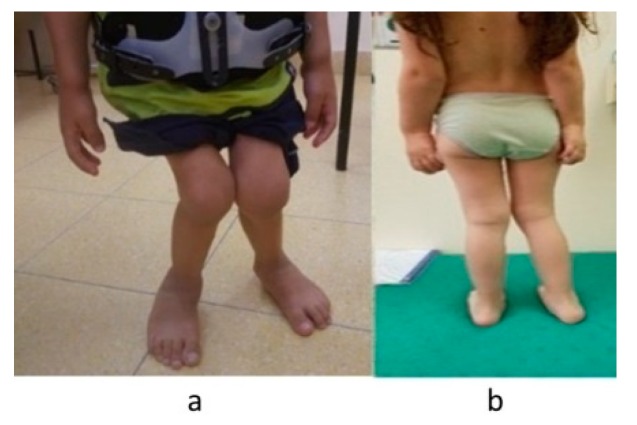
Lower limbs deformities: (**a**) patient 1 (72 months); (**b**) patient 2 (48 months).

**Figure 4:**
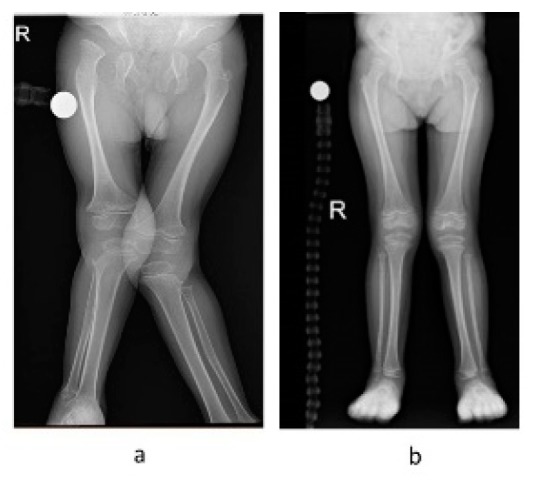
Radiographic pictures of the lower extremities of patients 1 and 2: (**a**) patient 1 (84 months); (**b**) patient 2 (48 months).

Both patients presented timely and typical skeletal changes of MPS IVA involving the spine (odontoid hypoplasia and kyphoscoliosis), chest cage deformities, upper extremities abnormalities (ulnar deviation and elbow valgus), and lower extremities abnormalities (hips dysplasia, genu valgus, calcaneal valgus, and pronated feet). The main differences between the siblings are the severity of the deformities and the stability of the cervical spine (see Figure 3 and Figure 4).

### 3.5. Pulmonology and Ear, Nose, and Throat (ENT) Manifestations

P1 presented effortful, noisy breathing and snoring since birth and through all follow-up years, which alternated with morning fatigue and daytime sleepiness. He has been under regular follow-up since the age of 42 months. Physical examinations revealed thoracic cage deformities with restrictive movement and enlarged adenoid tissue. P1 also suffered from recurrent bilateral severe otitis media, which required the insertion of ventilation tubes at the age of 66 months under anesthesia, resulting in partial improvement. P1 underwent multiple sequential respiratory and sleep tests for obstructive sleep apnea, which showed upper respiratory tract involvement with fair pulmonary functions (see Table 1). P1 had a single audiometry test at 5 years old, which showed mild hearing loss.

During the follow-up period, P2 had no respiratory symptoms or sleeping complaints, except for occasional snoring. A single pulmonary examination at the age of 48 months was normal. Baseline sleep and hearing evaluations were within a normal range.

### 3.6. Physical Function

The first evaluation of P1 was at 30 months of age, two years before ERT. The first evaluation of P2 was at 48 months of age, three years after ERT. Table 2 provides details of the patients’ physical function.

#### 3.6.1. Impairment Level

Both patients presented various limitations in their range of motion, endurance, balance, and protective reactions. However, P1’s impairments were considerably more severe in comparison to P2 (see Table 2).

#### 3.6.2. Mobility

Both patients presented mobility difficulties, with significant differences between siblings in severity level (i.e., performance level, use of assistive devices, and environmental adaptations) (see Table 2).

#### 3.6.3. Activities of Daily Living

At 48 months, P1 still needed support with offing and doffing, whereas P2 was independent and age-appropriate. For additional information, see Table 2.

In summary, during follow-up, both patients presented progression of limitations. However, in comparison to P1, P2’s functional level was better maintained and more closely resembled age-appropriate developmental milestones.

## 4. Discussion

This case study provides clinical evidence regarding the 4.5-year efficacy and safety of elosulfase alfa treatment for MPS IVA in two siblings. Moreover, this is a unique opportunity to describe the effect of ERT started at a relatively young age (<1 year) [26] as we compare two patients with identical genetic data and shared environmental conditions.

### 4.1. Safety and Compliance

Our study is consistent with the results of other clinical studies, showing acceptable compliance and an acceptable safety profile [23,26,33,34].

### 4.2. Growth

The study showed that ERT has a limited effect on growth. These results are consistent with studies comparing ERT effects on the growth of MPS IVA patients [28,35,36]. Moreover, P2’s growth curve demonstrates a major change in growth velocity around the age of two years, which is typical for MPS IVA patients. In addition, until that age (before one year of age), the growth of children with MPS IVA does not differ from the normal population [28]. Finally, it is important to note that the initiation of GH even before diagnosis did not change the course of growth development.

### 4.3. Clinical Course, and Orthopedic and Radiographic Assessments

Patients with MPS IVA typically need numerous orthopedic procedures throughout their lifetime [9,10,11]. Moreover, these patients are at high risk for anesthesia morbidity and mortality [37]. Both patients presented typical MPS IVA skeletal deformities. However, the remarkable difference between the two was the stability of the cervical spine, which is a common problem in MPS IVA. When present and untreated, it may have serious consequences [8,38,39,40]. Even though P1’s surgical intervention operation for C1–C2 fixation was successful, it influenced his overall developmental course. Unlike P1, P2 did not require any surgical intervention by the end of the follow-up at the age of 66 months, although she did present typical changes in the lower extremities. These findings are encouraging as children with MPS IVA do commonly require surgical intervention as young as 48 months of age [39].

Furthermore, in a 5-year period, P1 had two prolonged rehabilitation periods in a rehabilitation hospital followed by unceasing physiotherapy, occupational therapy, and later, psychotherapy. P2, except for a limited period of weekly hospital visits for ERT, had only outpatient clinic visits, and physical and occupational therapies at the kindergarten. Given the clinical course, it appears that relatively early ERT did not prevent orthopedic deformities; however, it decreased the rate of progression and possibly postponed the need for surgical interventions. Any lessening of the need for surgery is beneficial to the child’s and family’s quality of life, as surgery is a stressful event for both the child and their family.

### 4.4. Respiratory Function and Sleep Test

P1 presented moderate-to-severe obstructive sleep apnea prior to ERT initiation, which was normalized 4.5 years post-treatment. Considering the increased tendency of MPS IVA patients to have upper airway obstruction [8,38], it appears that ERT might be beneficial in reducing the severity of respiratory complications. As for P2, one can carefully assume that starting ERT at a relatively young age was beneficial in preventing typical respiratory problems. These results are consistent with a multicenter study including 20 patients with MPS IVA, which reported sustained improvements in respiratory function using elosulfase alfa [41]. In a more recent study, Kenth and colleagues [42] also showed that ERT in MPS type IVA might attenuate the natural progression of respiratory dysfunction, especially in oximetry tests. Another possible physiological mechanism for the attenuated respiratory problems observed in both P1 and P2 is the functional ability of the children, especially in P2. More specifically, at the end of the follow-up period, P2 was still not using a powered wheelchair and walked 400 m in the six-minute walk test. As active individuals show higher spirometric results [43], staying physically active may have a beneficial effect on respiratory function.

### 4.5. ENT Manifestations

Over 90% of patients with MPS IVA present ENT complications at an early age, even while being treated with ERT [44,45,46]. In the current study, considering both siblings’ findings, it appears that P1’s clinical course is consistent with the literature. In contrast, starting ERT at a younger age (before 1 year of age) might be helpful in preventing or lessening ENT involvement.

### 4.6. Physical and Functional Status

Both patients presented progression of limitations in physical and functional status. However, in comparison to P1, P2’s functional level was better maintained and more closely resembled age-appropriate developmental milestones. Overall, these findings do not suggest that ERT was useful in achieving significant skeletal changes, whereas it did have an effect on functional capacity, including endurance, balance, and activities of daily living. These findings are not surprising as ERT with elosulfase alfa has been shown to improve endurance and exercise capacity [20,35,47], partially due to improved respiratory function and oxygen utilization [13].

### 4.7. Study Limitations and Conclusions

The present study is subject to limitations. This is not a pre-designed clinical trial, and P1 presented a complicating spinal cord injury. Therefore, there was variability regarding the medical evaluations schedule. Next, this study did not have a single endpoint other than safety aspects of ERT. Finally, it is difficult to make definite conclusions based on a 4.5-year follow-up and on the two case studies without conducting a longer follow-up and a comparison to a control group. However, it is important to note that in comparison to P2, P1 did start ERT at a much older age and the experience presented in this study reflects what clinicians probably encounter in daily practice.

In conclusion, ERT with elosulfase alfa in two siblings with MPS IVA was well-tolerated. Despite the treatment starting at a young age, the disease still progressed but at a different rate. Therefore, our findings highlight the importance of starting ERT as early as possible. Moreover, although an early initiation of ERT did not prevent orthopedic and growth problems, relatively early ERT initiation might delay the need for early-on complex surgeries and can be beneficial in maintaining health, functional ability, and quality of life. Nonetheless, despite the benefits of early ERT initiation observed in the study, considering the study limitations and ERT’s limited efficacy in preventing or resolving the disease’s progression (e.g., development of typical skeletal changes of MPS IVA), the study results should be interpreted with caution and further studies on ERT and other novel potential treatments are necessary.

## Figures and Tables

**Table 1:** Patient 1’s respiratory function and sleep test.

Age	Oxygen Drop	Minimal Oxygen Saturation (%)	Apnea-Hypopnea Index	Obstructive Sleep Apnea	Forced Vital Capacity (%)	Forced Expiratory Volume in 1 Second (%)
48	74	66	8.4	Moderate to severe	75	80
54	45	78	5.5	Moderate	80	85
102	16	90	2.3	Normal	82	89

**Table 2:** Summary of patients 1 and 2—age of initiation of limitations in physical function.

Function	Age (Months)	Patient 1	Patient 2
Range of motion	30–78	Neck and chest brace	
	48	Elbow extension	Elbow extension
Strength	36	Progressive weakness in four limbs and trunk	Pelvic girdle weakness; moderate trunk weakness
Protective reactions and balance	36	Partial protection reactions; impaired static and dynamic balance	Mild impairment in balance
Sitting	84	Difficulty in sitting upright	Independent
Walking	36	Independent for short distances on even surfaces/indoors; supervision for walking outdoors; poor endurance.	
	48	Walking only indoors; outdoors—using toddler ride-on toy on even surfaces and for short distances	Independent with no assistance devices on even surfaces for short and long distances with postural compensations
	66	See aforementioned	Six-minute walk test—400 m (age-expected—573 m)
	102	Powered wheelchair	
Standing up	36	Gower’s sign/external support	Independent, partial Gower’s sign; postural compensations
Stairs	36–66	Assistance, non-reciprocating	Handrail support
Offing and doffing	48	Partially independent	Independent
Drinking and eating	66	Partially independent	Partially independent

## Abbreviations

MPS	Mucopolysaccharidosis
GAG	Glycosaminoglycan
GALNS	N-acetylgalactosamine 6-sulfatase
ERT	Enzyme replacement therapy
GH	Growth hormone
P1	Patient 1
P2	Patient 2
ENT	Ear, nose, and throat
