# Correction: Barak, S. et al. “Long-Term Outcomes of Early Enzyme Replacement Therapy for Mucopolysaccharidosis IV: Clinical Case Studies of Two Siblings”. *Diagnostics* 2020, *10*, 108

**DOI:** 10.3390/diagnostics10070480

**Published:** 2020-07-14

**Authors:** Sharon Barak, Yair Anikster, Ifat Sarouk, Eve Stern, Etzyona Eisenstein, Tamar Yissar, Nir Sherr-Lurie, Annick Raas-Rothschild, Dafna Guttman

**Affiliations:** 1 Department of Pediatric Rehabilitation, Edmond and Lily Safra Children’s Hospital, Chaim Sheba Medical Center, Ramat-Gan 5265601, Israel; Etzyona.Eisenstein@sheba.health.gov.il (E.E.); Tamar.Yissar@sheba.health.gov (T.Y.); Dafna.Gutman@sheba.health.gov.il (D.G.); 2 Kaye Academic College of Education, M.Ed. Programs, Beer-Sheva 8414201, Israel; 3 The Sackler School of Medicine, Tel Aviv University, Tel Aviv 6997801, Israel; Yair.Anikster@sheba.gov.il; 4 Edmond and Lily Safra Children’s Hospital, Chaim Sheba Medical Center, Ramat Gan 5265601, Israel; 5 Wohl Institute for Translational Medicine, Chaim Sheba Medical Center, Ramat Gan 5265601, Israel; 6 The National AT Center, Chaim Sheba Medical Center, Ramat Gan 5265601, Israel; Ifat.Sarouk@sheba.gov.il; 7 The Pediatric Pulmonology Unit, Chaim Sheba Medical Center, Edmond and Lilly Safra Children Hospital, Tel HaShomer, Ramat Gan 5265601, Israel; 8 Pediatric Endocrine and Diabetes Unit, Chaim Sheba Medical Center, Edmond and Lily Safra Children’s Hospital, Ramat-Gan 5265601, Israel; zipporaheve.stern@sheba.health.gov.il; 9 Pediatric Orthopedic Unit, Edmond and Lilly Safra Children Hospital, Chaim Sheba Medical Center, Ramat Gan 5265601, Israel; Nir.Sherr@sheba.health.gov.il; 10 Institute of Rare Diseases, Edmond and Lily Safra Children’s hospital, Chaim Sheba Medical Center, Ramat Gan 5265601, Israel; Annick.Rothschild@sheba.health.gov.il; 11 The Sackler Faculty of Medicine, Tel Aviv University, Tel Aviv postcode Israel, Ramat Aviv 69978, Israel

The authors wish to make the following correction to this paper [1]: 

In the first paragraph of the Results section: “P1 and P2 started ERT with elosulfase alfa (VIMIZIM^®^ by BioMarin©) 5 months post-diagnosis at age 4.5 years and 11 months, respectively, with an intravenous dose of 1.0 mg/kg/week.” The value of the dose is 1.0 mg/kg/week but should be corrected to 2.0 mg/kg/week. 

These changes have no material impact on the conclusions of our paper. We apologize to our readers.
